# Plasma versican and plasma exosomal versican as potential diagnostic markers for non-small cell lung cancer

**DOI:** 10.1186/s12931-023-02423-4

**Published:** 2023-05-31

**Authors:** Wenjing Chang, Jichao Zhu, Dianyu Yang, Anquan Shang, Zujun Sun, Wenqiang Quan, Dong Li

**Affiliations:** 1 Department of Laboratory Medicine, Shanghai Tongji Hospital, School of Medicine, Tongji University, Shanghai, 200065 China; 2 Department of Laboratory Medicine, Huzhou Central Hospital, Affiliated Central Hospital of Huzhou Normal University, Huzhou, 313003 China

**Keywords:** NSCLC, Versican, Exosomes, Metastasis, Diagnostic value

## Abstract

**Background and aims:**

This study aimed to investigate the expression of plasma versican and plasma exosomal versican in non-small cell lung cancer (NSCLC) and its correlation with clinicopathological features, and to evaluate its diagnostic performance in NSCLC and its predictive function for NSCLC incidence and metastasis risk.

**Materials and methods:**

There were 110 instances of NSCLC, 42 cases of benign lung disease, and 55 healthy controls from September 2018 to October 2020 at Tongji Hospital Affiliated to Tongji University. Blood was collected and plasma was separated before surgery, and plasma exosomes were extracted by ExoQuick kit. Morphological and molecular phenotype identification of exosomes was performed by transmission electron microscopy, Nanosight particle tracking analysis, and western blotting. Plasma versican and plasma exosomal versican were detected in all subjects to assess their expression levels and diagnostic value in NSCLC. Clinicopathological data were collected to explore correlations between abnormal plasma versican and plasma exosomal versican expression and clinicopathological parameters. Receiver operating characteristic (ROC) curve was used to judge its diagnostic performance in NSCLC, and binary logistic regression analysis was used to predict the risk of NSCLC incidence and metastasis.

**Results:**

Plasma versican and plasma exosomal versican expression in NSCLC patients was significantly upregulated and was significantly higher in T3 + T4 patients compared with T1 + T2 patients (P < 0.05); the levels of plasma versican and plasma exosomal versican were positively correlated with lymph node metastasis, distant metastases (e.g., brain, bone), and mutation(e.g., EGFR,ALK)in NSCLC patients (all P < 0.05). Furthermore, ROC curve analysis showed that plasma versican and plasma exosomal versican had higher AUC values than NSE, CYFRA21-1, and SCC, and better diagnostic performance in NSCLC patients. However, the AUC and diagnostic performances of plasma versican and plasma exosomal versican in advanced-stage NSCLC patients were not shown to be significantly better than CEA. The results of binary logistic regression analysis showed that high levels of plasma exosomal versican had higher predictive value for lung cancer incidence, while high levels of plasma versican had higher predictive value for lung cancer metastasis.

**Conclusion:**

Our findings showed that plasma versican and plasma exosomal versican might be potential diagnostic markers for NSCLC. High plasma exosomal versican expression can be used as a predictor of NSCLC risk and high plasma versican expression can be used as a predictor of NSCLC metastasis risk.

**Supplementary Information:**

The online version contains supplementary material available at 10.1186/s12931-023-02423-4.

## Introduction

Lung cancer is the most common cancer-related fatality worldwide, accounting for almost a quarter of all cancer-related fatalities. Non-small cell lung cancer (NSCLC) is the most prevalent lung cancer subtype, accounting for approximately 80% of the overall incidence of lung cancer [1]. Currently, because of the lack of specific diagnostic markers for NSCLC, and the occult early symptoms, often accompanied by metastasis, most of the patients were in the advanced stage when they were found. The therapeutic landscape of advanced NSCLC has significantly improved with the advent of immunotherapy and targeted therapies, and they provide “effective” albeit non-durable control of the disease. However, the poor 5-year survival rate of advanced NSCLC patients has not significantly improved. Lung cancer still accounts for more than 80% of all deaths from cancer. Lung cancer accounts for more than 80% of all deaths from cancer [2, 3]. The development of NSCLC is dependent not only on genetic alterations in tumor cells, but also on changes in the tumor microenvironment, which includes stroma, blood vessels, infiltrating inflammatory cells, and other components [4, 5]. The extracellular matrix, in addition to its biological importance as a component of the tumor microenvironment, also plays a crucial role in the progression of cancer [6].

Versican is a high molecular weight chondroitin sulfate glycoprotein that is a member of the lecican proteoglycan family and is a critical component of the extracellular matrix [7]. Versican is encoded by *VCAN gene*, which is located on chromosome 5q12–14 of the human genome. *VCAN* has 15 exons and is more than 90–100 kb in length, with the first exon being the longest. Versican protein comprises three critical domains: the G1 domain at the N end of the peptide chain, the G3 domain at the C end of the peptide chain, and the chondroitin sulfate chain that connects the G1 and G3 domains. The G1 domain comprises an immunoglobulin (IG)-like motif, as well as two hyaluronic acid-binding proteoglycan(HABr) repeats that are essential for cell adhesion. In the G3 domain, two Pro-epidermal growth factor-like growth factor (EGFR) repeats are found in tandem with a carbohydrate recognition domain and a complement binding protein-like motif. It has been shown that versican can be present in at least four different sources in the tumor microenvironment, including cancer cells, tumor-associated myeloid cells, tumor-infiltrating lymphocytes, and stromal cells [8].

In the tumor microenvironment, versican interacts with a variety of cell types by binding to integrins and integrin receptors, as well as other extracellular matrix components associated with the cell surface. It is involved in the adhesion of tumor cells and their proliferation, migration, and angiogenesis [9, 10]. Versican has also been shown to have a crucial role in the transformation and advancement of malignant tumors, with higher expression of versican in a range of malignant tumors being related with cancer recurrence and poor prognosis [11]. Cancer cells have been identified as a prominent source of secreted versican in several cancer types. In a mouse model of Lewis lung cancer(LLC), versican secreted by tumor cells promoted lung, liver, and adrenal cancer metastasis [12]. This process is dependent on Toll Like Receptor 2(TLR2)-mediated activation of bone marrow-derived macrophages and the involvement of tumor necrosis factor. Furthermore, stromal cells are also a source of Versican in the tumor microenvironment, and the high expression of Versican from the matrix was associated with the increased expression of Hyaluronic acid(HA), and the expression level of HA was positively correlated with the expression level of cell surface receptor CD44 [9]. A subtype of versican, versican V1, has been shown to be an important tumor growth-promoting and metastasis-promoting factor that induces the proliferation and development of ovarian cancer by regulating the inflammatory response in the tumor microenvironment [13]. Activation of myeloid cells such as macrophages to release the inflammatory cytokine cathelicidin (HCAP18/LL-37, Human cationic antimicrobial protein 18) and induce proliferation of lung cancer cells has also been demonstrated [14]. Versican V1 is secreted by tumor cells in the tumor microenvironment. Generalized expression of versican has been observed in a wide range of malignant tumors, including glioma, liver tumors, and lung tumors, as well as breast cancer, prostate tumors, stomach tumors, and bowel tumors [15]. Versican also plays a critical role in the malignant transformation and progression of tumors.

Liquid biopsy is a repeatable, non-invasive procedure that may be used to diagnose tumors in their early stages [16]. To date, there are few plasma indicators for lung cancer. Clinical tumor markers such as neuron-specific enolization enzyme (NSE), carcinoembryonic antigen (CEA), cytokeratin 19 (CYFRA21-1), and squamous cell carcinoma antigen (SCC) have demonstrated low sensitivity and specificity in the diagnosis of early-stage lung cancer, resulting in irreparable harm to the patient’s clinical diagnosis, treatment, and outcome. Thus, it is critical to identify novel tumor markers for liquid biopsy that are both sensitive and specific.

Recently, exosome detection has become one of the most popular liquid biopsy methods [17]. Exosomes contain DNA, microRNA, long-chain non-coding RNA, and several types of proteins, all of which are implicated in the malignant progression of tumors [18]. Exosomes have a distinct membrane structure that protects their contents from the effect of the microenvironment outside the membrane and allows the exosomes to remain in a stable condition for extended periods. Furthermore, exosomes and the contents of plasma exosomes have emerged as novel and reliable biomarkers that may be used to aid the early diagnosis and prognostic assessment of tumors [19, 20]. In the context of the tumor microenvironment, the molecular properties and biological activities of versican suggest that it has great potential as a molecular marker for tumor diagnosis and prognosis. However, although many studies have focused on the use of immunohistochemical (IHC) labeling of versican to detect malignancies [21], there has been no report on the expression of versican or its diagnostic utility in lung cancer plasma or lung cancer exosomes. There is a need to investigate the expression of plasma versican and plasma exosomal versican in non-small cell lung cancer (NSCLC) and its correlation with clinicopathological features, and to evaluate its diagnostic performance in NSCLC and its predictive function for NSCLC incidence and metastasis risk.

## Materials and methods

### Patients and clinical information

We collected 110 lung cancer patients who were hospitalized in Tongji Hospital Affiliated to Tongji University from September 2018 to October 2020, 42 patients with benign lung disease, and 55 healthy controls who came to the hospital during the same period as matched groups for this study. Lung cancer was surgically removed, and diagnosis was confirmed by pathology, cytology, and histology. The 8th edition of the Union for International Cancer Control utilized the tumor-lymph node-metastasis (TNM) classification of lung cancer to stage NSCLC. Exclusion criteria were inadequate imaging data, clinical stage unknown, chest computed tomography(CT), abdominal B-ultrasound, head Magnetic Resonance Imaging(MRI), bone scan identified as other malignant tumors, prior adjuvant treatment such as radiation or chemotherapy, and an inability to finish the research procedure. The control group of benign lung diseases included chronic pulmonary inflammation, pulmonary nodules, pulmonary fibrosis, chronic obstructive pulmonary disease, etc., the inclusion criteria of the patients were: 1) no history of lung cancer and no other cancer diagnosed or treated within 5 years; 2) no radiotherapy, immunotherapy or surgery for the lung; 3) no autoimmune disease; 4) no history of severe liver or renal dysfunction. At the same time, the healthy people who were examined in our hospital were selected as the healthy control group, the inclusion criteria were: 1) routine physical examination、laboratory examination and imaging examination were normal; 2) no history of cancer; 3) no severe underlying diseases such as endocrine disease, cerebrovascular disease, chronic liver disease, chronic kidney disease, liver and kidney dysfunction. The ethics committee of Tongji Hospital affiliated with Tongji University approved this study [(Tong) Lunxun-KYSB-2018-(087)].

After fasting from 22:00 the previous evening, patients’ peripheral venous whole blood was collected at 08:00 the following morning. Used EDTA-K2 anticoagulant tube and pro-coagulant tube to collect 5ml of venous blood from the patients. The whole process requires aseptic operation, centrifuged at 2000*×g* for 10 min, and plasma/serum was obtained within 30 minutes after sample collection. Removed the supernatants and stored at a low temperature of -80℃ to avoid freezing and thawing. In this study, the four tumor markers neuron specific enolization enzyme (NSE), carcinoembryonic antigen (CEA), cytokeratin 19 (CYFRA21-1), and squamous cell carcinoma antigen (SCC) were investigated and analyzed by ECL800 electrochemiluminescence instrument and its supporting reagents(Roche,Germany). In order to ensure the accuracy of the experiment, the supporting calibrators, detection reagents and quality control products provided by the manufacturer were uniformly used in the experiment process.

### Plasma exosome isolation

An ExoQuick kit (Bestbio, Shanghai, China) was used to extract human plasma exosomes according to the manufacturer’s instructions. Briefly, after the frozen plasma samples were equilibrated to room temperature, samples were centrifuged at 3000 ×g for 15 min to remove residual cells and cell debris. Then 250 µl supernatant was removed to a new tube, to which 63 µl ExoQuick reagent was added. The tube was thoroughly mixed and incubated at room temperature for 30 min. This was followed by centrifugation at 1500 ×g for 30 min, after which the supernatant was discarded. This step was repeated for another 5 min, and the supernatant was discarded again. Finally, the remaining pellet was deemed as the exosome fraction.

### Transmission electron microscopy

A 20-µl exosome aliquot suspension was placed onto a 200-mesh carbon coated copper grid for 2 min. After excess liquid was removed using filter paper, the grid was negatively stained with 3% tungsten phosphate solution at room temperature for 3 min. The copper mesh was washed five times in double distilled water and allowed to dry naturally at room temperature. The sample was observed and photographed by transmission electron microscopy (TEM) (Thermo–Fisher, Waltham, MA, USA).

### Nanosight particle tracking analysis (NTA)

To identify the size distribution and concentration of isolated particles, a NanoSight LM10 system (Malvern Instruments Ltd. Malvern, UK) was used to detect the diluted exosomes. First, the sample detection slot was cleaned with Duchenne phosphate buffer without any nanoparticles. Then the exosome samples were diluted with Duchenne phosphate buffer 10 times and thoroughly mixed. The concentration and particle size of exosomes samples were determined by the NanoSight LM10 system software.

### Western blotting analysis

The BCA method was used to quantify exosome protein, and the concentration of the exosome protein was adjusted according to the quantitative results. Diluted exosomes were added to sodium dodecyl sulfate buffer and boiled for western blot analysis. In this study, 10% separating gel was used. The gel and electrodes were assembled in the SDS-PAGE chamber. Next, 20 µl sample per well was loaded onto a 10% acrylamide gel for electrophoresis running at 120 V until the bromophenol blue front ran out of the gel. Proteins on the gels were transferred to nitrocellulose or polyvinylidene fluoride membranes and detected by immunoblotting. Primary antibodies against CD54, HSP70, Flotillin-1, and GM130 were obtained from Cell Signaling Technology (Danvers, Massachusetts, USA). Secondary antibodies were rabbit-anti-mouse (Dako, Carpinteria, CA, USA) or HRP-conjugated goat anti-rabbit (Santa Cruz) antibodies.

### Enzyme-linked immunosorbent assay (ELISA) analysis

Plasma versican and plasma exosomal versican were detected according to the method recommended in the ELISA kit instructions (Cloud-Clone CORP., Wuhan, China). The plasma sample from which residual cells and cell debris had been removed was diluted with 1×PBS at an appropriate multiple. The exosome pellets were lysed with 100 ml RIPA lysis buffer on ice for 30 min, shaken and mixed thoroughly, and diluted with 1×PBS. Then 100 µl each of the standard, blank control, diluted serum, and exosomal samples of each group were added to the versican antibody-coated microtiter plate and incubated at 37 °C for 60 min. The liquid was then removed from the microtiter plate and patted dry, and solution A was added. The plate was then incubated at 37 °C for 60 min and washed three times before the addition of solution B and a further incubation at 37 °C for 30 min. The plate was washed again five times and 90 µl substrate was added and incubated at 37 °C for 15 min in the dark. Then 50 µl stop solution was added and the plate was measured immediately at 450 nm.

### Statistical analysis

SPSS 25.0 was used for the statistical analysis. Kolmogorov–Smirnov test was used for the measurement of data for normal test analysis. The standard deviation of normally distributed data was expressed as ‾x ± standard deviation (SD). A *t*-test (data conforming to normal distribution and homogeneity of variance) or Wilcoxon rank sum test (non-conformity of normal distribution and homogeneity of variance) was performed to compare the two groups. For 3 groups of multiple comparisons, a test was performed using one-way ANOVA. Numeration data were analyzed using the χ2 test. The skew distribution measurement data were displayed as the median (P50). Independent sampling was used to compare skewed distribution measurement data using the Kruskal–Wallis *H* test.

GraphPad Prism8.0 software was used to create the receiver operating characteristic (ROC) curve of each indicator and to combine the test to determine the sensitivity, specificity, cutoff value, Youden index, positive predictive value (PPV) and negative predictive value (NPV) of each indicator in lung cancer patients. The area under the curve (AUC) was used to evaluate the accuracy of the test. Spearman’s rank correlation coefficient was used to analyze the correlation between the levels of plasma versican or plasma exosomal versican and the clinical parameters in NSCLC patients. Binary logistic regression analysis was applied to evaluate the predictive risk value of the levels of plasma versican and plasma exosomal versican for NSCLC or NSCLC patients with metastasis using median (P50) as cutoff points, respectively. It was also used to calculate the values of the single-factor- and multifactor-adjusted odds ratios (AORs) and 95% confidence intervals (CI) according to maximum likelihood estimates. *P* < 0.05 was considered statistically significant.

## Results

### Patient characteristics

A total of 110 patients with NSCLC were recruited for this study, comprising 52 men and 58 women aged (65 ± 10) years, all of whom were histopathologically diagnosed with NSCLC and had not had any previous therapy. A total of 42 patients with benign lung disease were selected as the benign control group at Tongji Hospital during the same period, comprising 21 men and 21 women aged (63 ± 12) years. Fifty-five healthy subjects were selected as the normal control group during the same period, comprising 29 men and 26 women aged (60 ± 14) years. There were no statistically significant differences in the distribution of age, sex, and smoking status between the NSCLC, benign lung disease, and healthy control groups (*P* > 0.05) (Table 1).

**Table 1:** Characteristics of the enrolled patients

Characteristics	NSCLC	Benign lung diseases	Healthy controls	*P* value
Total number	110(%)	42(%)	55(%)	
**Age(years)**				
≤ 60	43(39.1)	17(40.5)	23(41.8)	*P >* 0.05^*^
> 60	67(60.9)	25(59.5)	32(58.2)	
**Gender**				
Male	52(47.3)	21(50.0)	29(52.7)	*P >* 0.05^*^
Female	58(52.7)	21(50.0)	26(47.3)	
**Smoking status**				
Former smoker and current	48(43.6)	16(38.1)	20(36.4)	*P >* 0.05^*^
Never	62(56.4)	26(61.9)	35(63.6)	
**Tumor subtype**				
ADC	94(85.5)	/	/	
SCC	16(14.5)			
**TNM stage**				
I + II	59(53.6)	/	/	
III + IV	51(46.4)			
**Lymph node metastasis**				
Yes	50(45.5)	/	/	
No	60(54.5)			
**Distal metastasis (Brain, bone…)**				
Yes	24(21.8)	/	/	
No	86(78.2)			
**Chemotherapy**				
Yes	46(41.8)	/	/	
No	64(58.2)			
**Recurrence**				
Yes	7(6.4)	/	/	
No	103(93.6)			
**Mutation(EGFR,ALK…)**				
Yes	42(38.2)	/	/	
No	68(61.8)			

Abbreviations: ADC, adenocarcinoma; SCC, squamous cell carcinoma. *P > 0.05 indicates NSCLC group vs. benign lung diseases group; NSCLC group vs. healthy control group; benign lung diseases group vs. healthy control group. There were no significant differences (χ^2^ test)

### Characterization of exosomes isolated from plasma

**Fig. 1:**
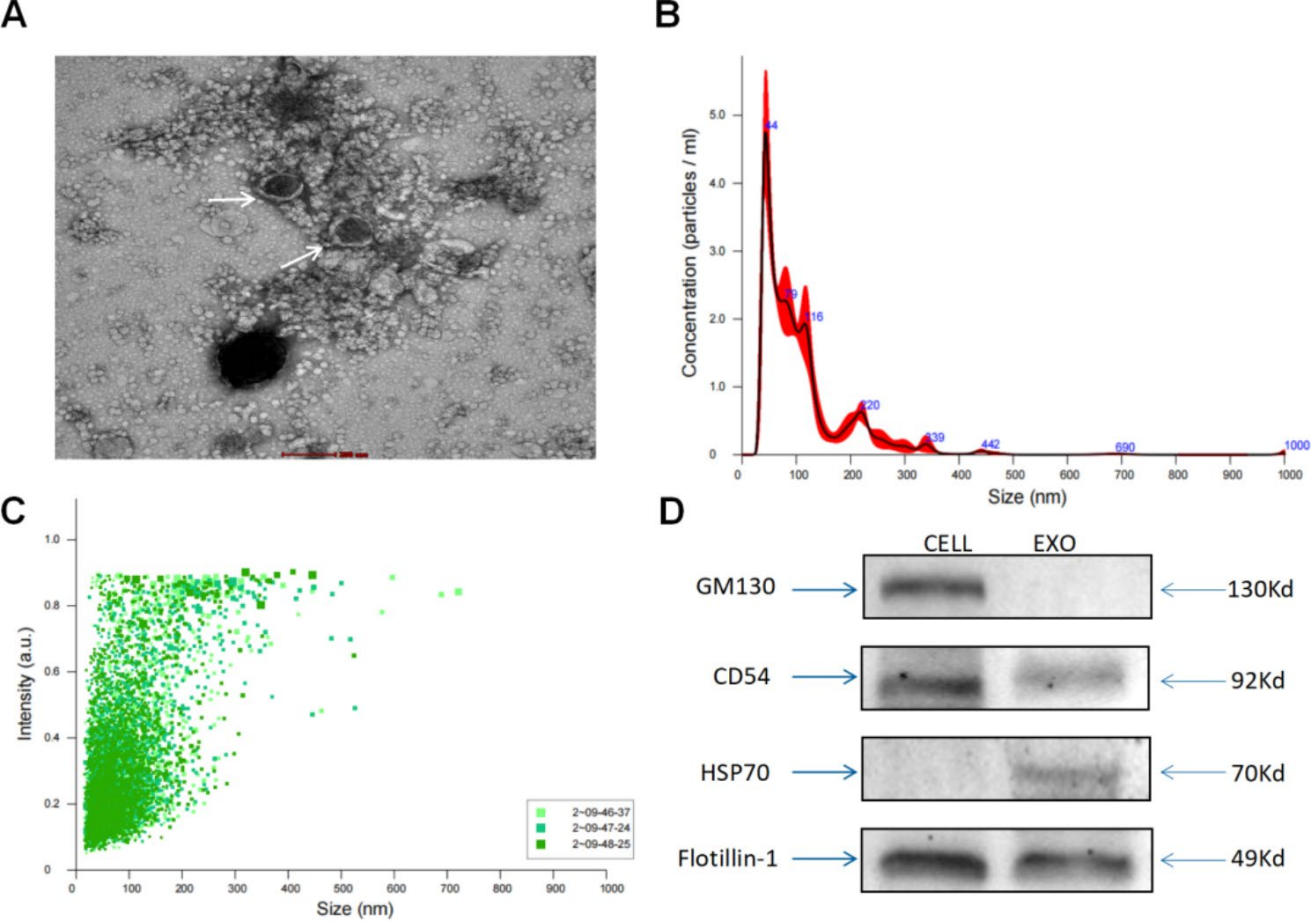
Characterization of exosomes isolated from plasma. (**A**) Transmission electron microscopy (TEM) showing the external features of exosomes isolated from plasma. (**B,C**) Nanoparticle tracking analysis (NTA) demonstrated the size distribution and concentration of exosomes isolated from plasma. (**D**) Western blotting analysis of characteristic markers of exosomes, including CD54, HSP70, and Flotillin-1, with GM130 as a negative control protein

An ExoQuick kit was used to extract all plasma samples. Nanoparticles were examined by NTA and TEM, as well as three antibody markers of extracellular vesicles, to confirm the kit’s efficacy in separating exosomes. Exosome extracts were round or quasi-round vesicles of approximately 100 nm in diameter with an intact envelope and a clear background under TEM. No cell pieces were observed (Fig. 1A). The peak size of patient plasma exosomes was between 40 and 150 nm, the greatest distribution peak was (109.5 ± 3.7) nm, and the concentration was (3.13 × 10^12^±3.92 × 10^10^) particles/ml according to NTA data (Fig. 1B C). Western blot examination revealed significant bands of exosomes markers CD54, HSP70, and Flotillin-1 in human plasma exosomes deposits (Fig. 1D). These findings validated the integrity and purity of the exosomes.

### Expression levels of plasma versican and plasma exosomal versican

**Fig. 2:**
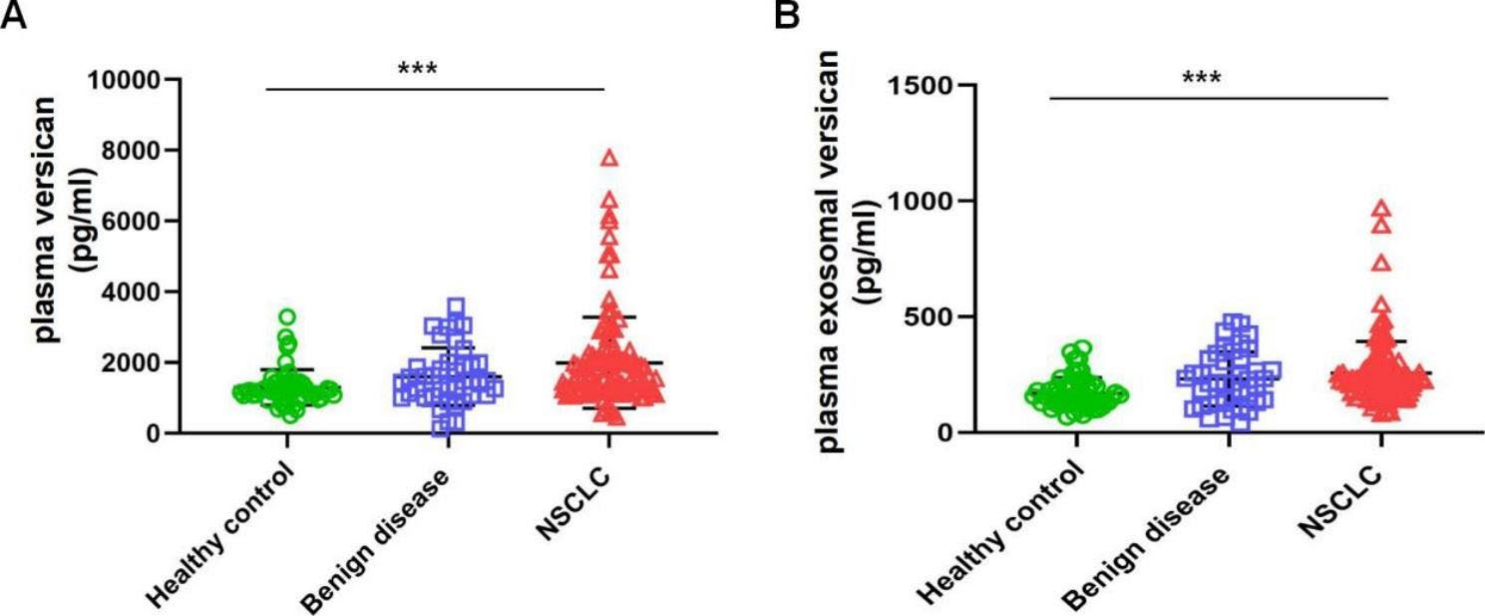
Expression levels of plasma versican(**A**) and plasma exosomal versican(**B**) in the three groups of subjects.(***P < 0.001)

As shown in Fig. 2A, plasma versican levels in the NSCLC group were greater than those in the benign disease and healthy control groups, and the difference was statistically significant (one-way ANOVA, F = 8.592, *P* < 0.001). Similarly, as shown in Fig. 2B, we found that the levels of plasma exosomal versican in the NSCLC group were greater than those in the benign disease and healthy control groups, and the difference was statistically significant (one-way ANOVA, F = 9.907, *P* < 0.001).

### Plasma versican and plasma exosomal versican levels at different NSCLC stages

**Fig. 3:**
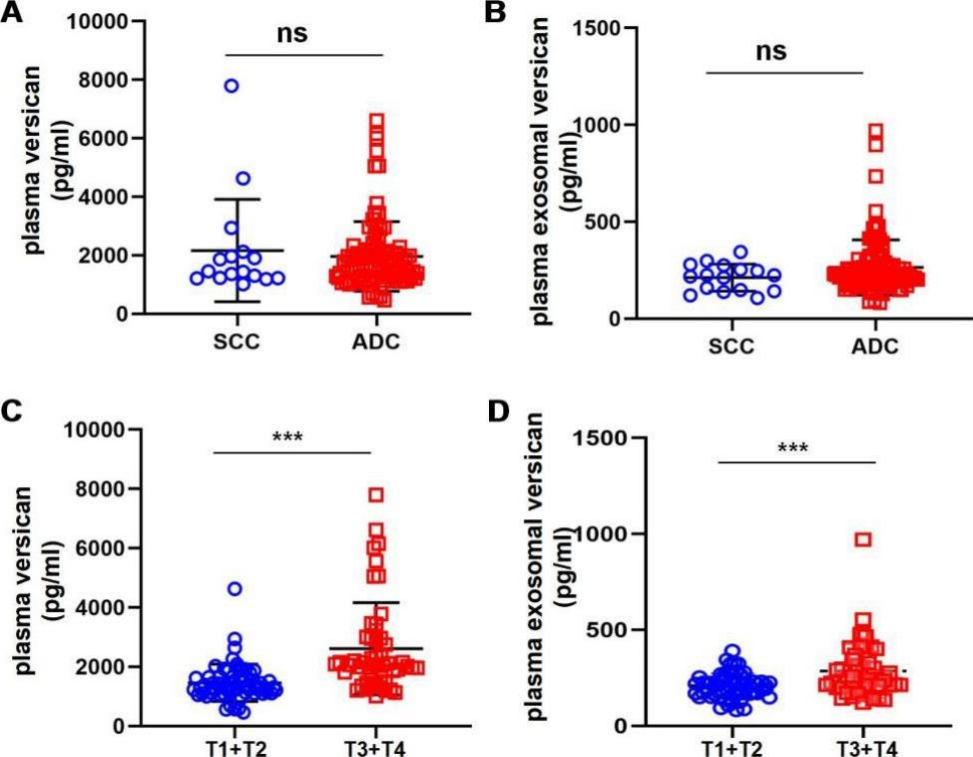
Plasma versican and plasma exosomal versican expression levels at different types of NSCLC or different NSCLC stages. (**A**) Plasma veriscan expresses no difference in SCC and ADC two types of NSCLC. (**B**) Plasma exosomal veriscan expresses no difference in SCC and ADC two types of NSCLC. (**C**) Plasma versican expression was significantly higher in T3 + T4 patients compared with T1 + T2 patients in NSCLC(***P < 0.001). (**D**) Plasma exosomal veriscan expression was significantly higher in T3 + T4 patients compared with T1 + T2 patients in NSCLC(***P < 0.001) (ADC, adenocarcinoma; SCC, squamous cell carcinoma)

ADC and SCC were the two most common forms of NSCLC in this study. We examined plasma versican levels in these two types of NSCLC and found no statistically significant difference (t = 0.582; *P* > 0.05; Fig. 3A), and there was no statistically significant difference between the two types of lung cancer (t = 1.426; *P* > 0.05; Fig. 3B). We also investigated plasma versican levels in NSCLC patients at different TNM stages. According to Fig. 3C, plasma versican expression levels were significantly higher in T3 + T4 patients compared with T1 + T2 patients (t = 5.212; *P* < 0.001); similarly, plasma exosomal versican levels were higher in T3 + T4 patients compared with T1 + T2 patients, and the difference was statistically significant (t = 3.715; *P* < 0.001; Fig. 3D).

### Correlation between plasma versican or plasma exosomal versican and clinical parameters in NSCLC patients

**Fig. 4:**
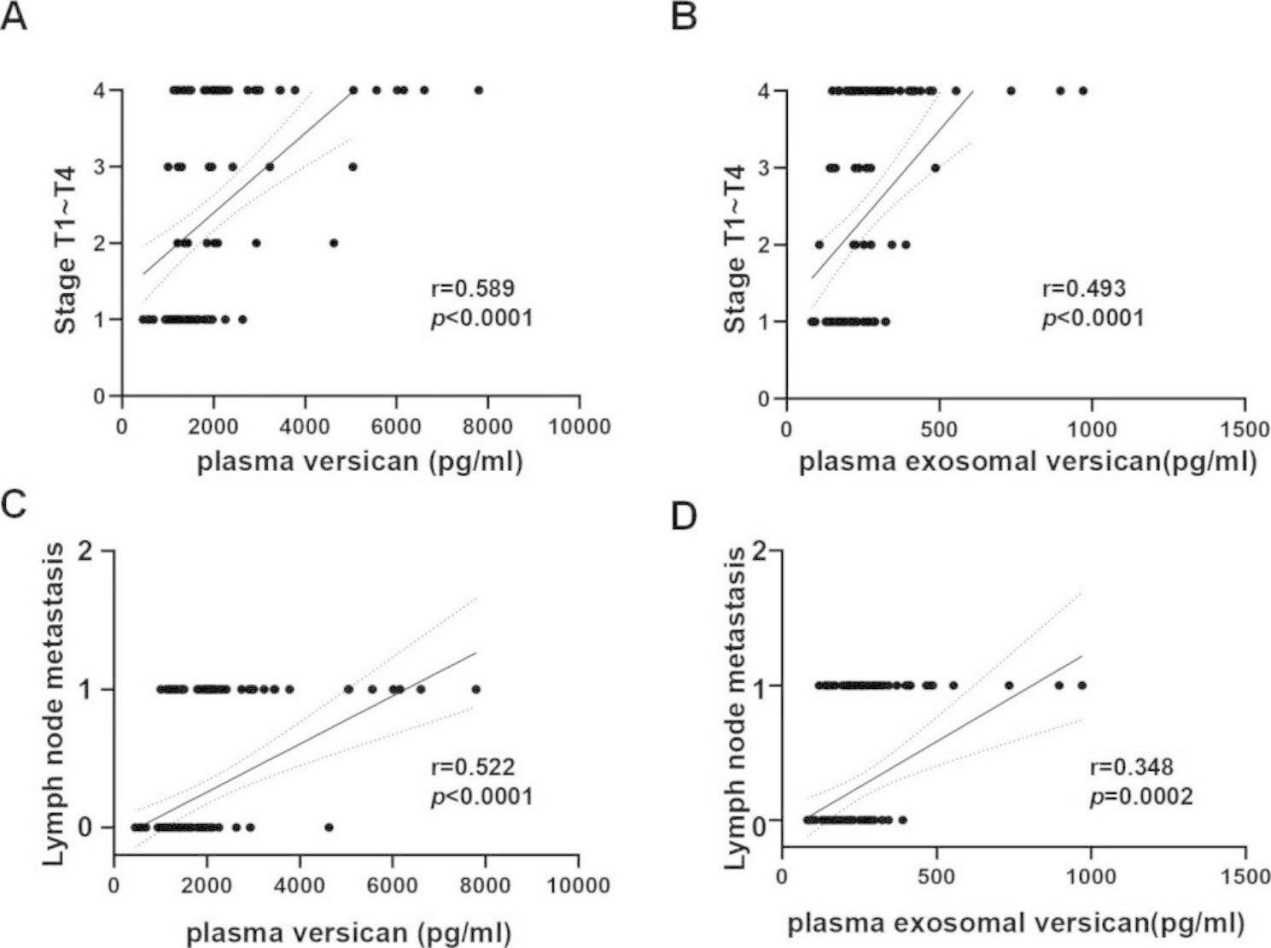
Correlation between plasma versican or plasma exosomal versican and clinical parameters in NSCLC patients. (**A**)Plasma versican or (**B**) plasma exosomal veriscan expression level was significantly positively correlated with T stage of NSCLC patients(*P* < 0.001). (**C**)Plasma versican or (**D**) plasma exosomal veriscan expression level was significantly positively correlated with Lymph node metastasis of NSCLC patients(*P* < 0.001)

We investigated potential correlations between plasma versican or plasma exosomal versican levels and clinical parameters in NSCLC patients. As shown in Fig. 4A, the levels of plasma versican were positively correlated with TNM stage (T1–T4) (*r* = 0.589, *P* < 0.0001) and lymph node metastasis (N = 0 or N ≥ 1) (*r* = 0.522, *P* < 0.0001; Fig. 4C). Similarly, plasma exosomal versican in NSCLC patients was positively correlated with TNM stage (*r* = 0.493, *P* < 0.0001; Fig. 4B) and lymph node metastasis (*r* = 0.348, *P* = 0.0002; Fig. 4D).

**Fig. 5:**
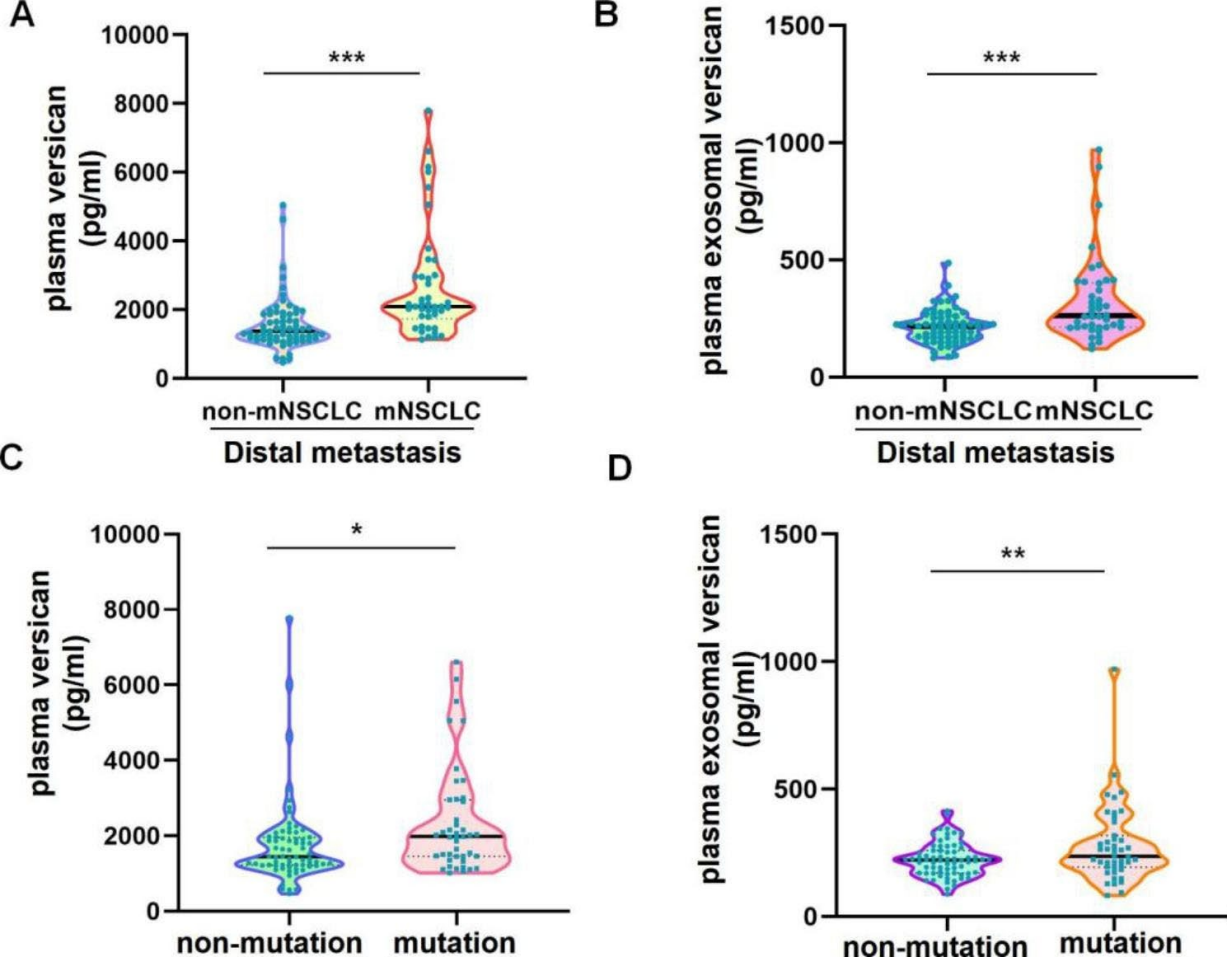
Differences between plasma versican or plasma exosomal versican levels and distant metastasis (e.g., brain, bone) or mutation(e.g., EGFR,ALK)in NSCLC patients. (**A**)Plasma veriscan expression level was significantly elevated in NSCLC patients with distant metastasis(***P < 0.001). (B)Plasma exosomal veriscan was significantly elevated in NSCLC patients with distant metastasis(***P < 0.001). (**C**)Plasma versican expression was significantly higher in mutation patients compared with non-mutation patients in NSCLC(*P < 0.05). (**D**) Plasma exosomal veriscan expression was significantly higher in mutation patients compared with non-mutation patients in NSCLC(**P < 0.01)

We proceeded to explore whether there were any correlations between plasma versican or plasma exosomal versican levels and distant metastases (e.g., brain, bone) or mutation(e.g., *EGFR*, *ALK*) in NSCLC patients. Because of the presence of distant metastasis (mNSCLC), the levels of versican protein expression in plasma were substantially higher in the distant metastasis group (mNSCLC) compared with the non-distant metastasis group (non-mNSCLC) (t = 4.902; *P* < 0.001; Fig. 5A). Meanwhile, statistically significant variations in plasma exosomal versican levels were observed between NSCLC patients with and without distant metastasis (non-mNSCLC vs. mNSCLC, t = 4.438; *P* < 0.001; Fig. 5B). Moreover, we discovered that plasma versican levels were significantly greater in the mutation group(e.g., *EGFR*, *ALK*) of NSCLC than in the non-mutation group (t = 2.486; *P* = 0.014; Fig. 5C). However, the levels of versican in plasma exosomal fractions were increased in the mutation group of NSCLC and the difference was statistically significant (t = 2.625; *P* = 0.010; Fig. 5D).

### Diagnostic performance of lung cancer markers NSE, CEA, CYFRA21-1, SCC, plasma versican, and plasma exosomal versican in NSCLC patients

**Fig. 6:**
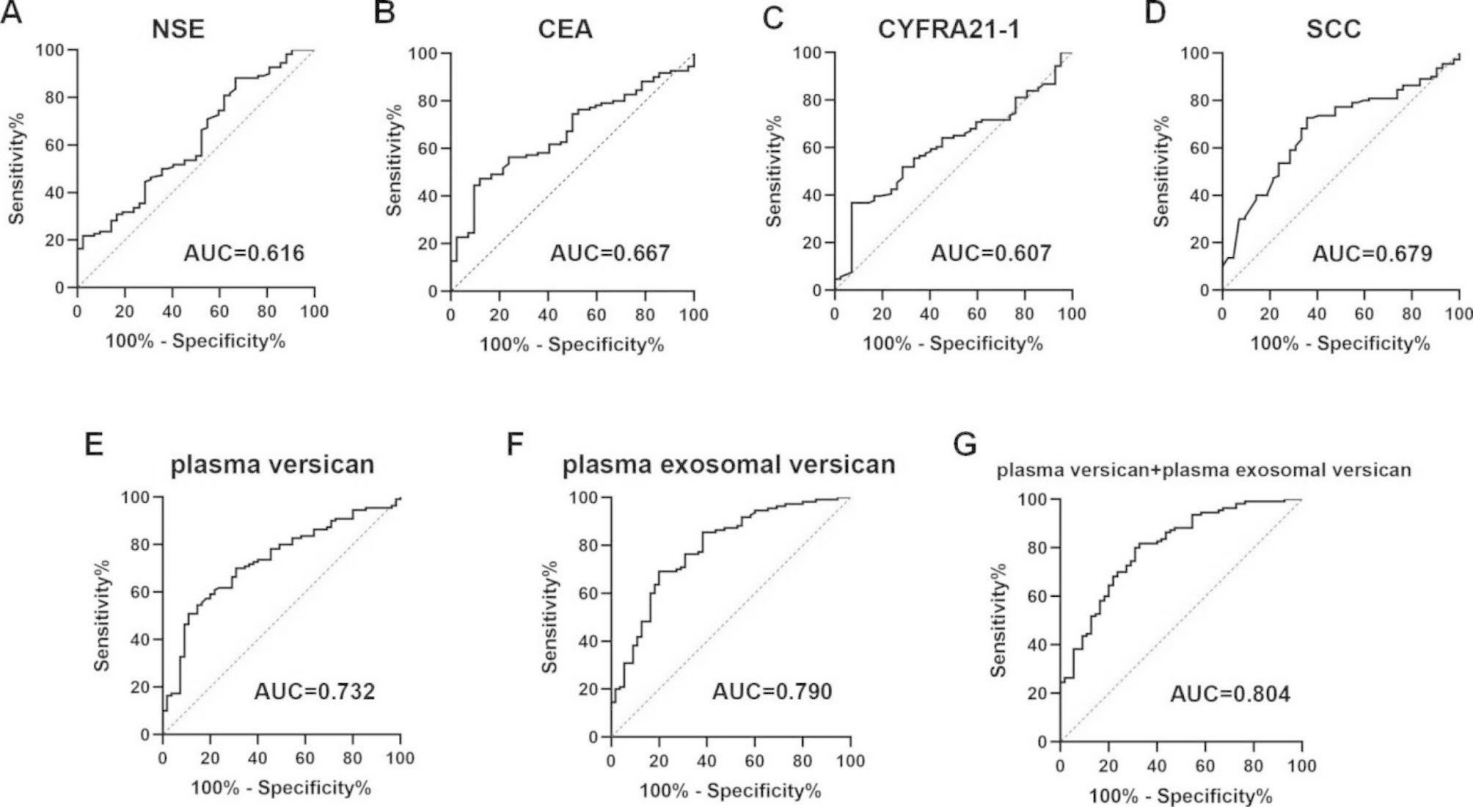
Diagnostic performance of lung cancer markers NSE, CEA, CYFRA21-1, SCC, plasma versican, and plasma exosomal versican in NSCLC patients

GraphPad Prism was used to draw the receiver operating characteristic (ROC) curves for the lung cancer markers NSE, CEA, CYFRA21-1, SCC, plasma versican, plasma exosomal versican, and the combination. According to Fig. 6A–G, the AUC of plasma versican was 0.732 when the cutoff value was1284.00 pg/ml, and the sensitivity, specificity, PPV, and NPV were 70.00%, 69.09%, 71.97%, and 67.01%, respectively. As an example, when the cutoff value was 166.30 pg/ml, the AUC of plasma exosomal versican was 0.790, and the sensitivity, specificity, PPV, and NPV were 85.4%, 61.82%, 71.74%, and 78.93%. Assuming a sensitivity of 80.00% and a specificity of 69.09%, the AUC of plasma versican + plasma exosomal versican was 0.804, and the combined detection sensitivity and specificity, as well as its PPV and NPV was 80.00% (Table 2). Following the results in Fig. 6; Table 2, plasma versican and plasma exosomal versican had higher AUC values than NSE, CYFRA21-1, and SCC, and had better diagnostic performance than NSE, CYFRA21-1, and SCC, and their diagnostic performance was superior to NSE, CEA, CYFRA21-1, and SCC. Undoubtedly, the AUC value of combined detection was higher, and the diagnostic performance was improved.

**Table 2:** Diagnostic performance of lung cancer markers, plasma versican, and plasma exosomal versican in NSCLC patients

Indicators	Youden index	Cutoff	AUC	AUC 95%CI	Sensitivity	Specificity	PPV(%)	NPV(%)
NSE	0.1598	12.89	0.616	0.5166–0.7146	44.55	71.43	63.88	53.18
CEA	0.3537	5.835	0.667	0.5782–0.7551	47.27	88.10	81.83	59.57
CYFRA21-1	0.2332	1.965	0.607	0.5120–0.7013	51.89	71.43	67.32	56.70
SCC	0.3702	1.310	0.679	0.5882–0.7691	72.73	64.29	69.79	67.52
plasma versican	0.3909	1284	0.732	0.6525–0.8104	70.00	69.09	71.97	67.01
plasma exosomal versican	0.4727	166.3	0.790	0.7157–0.8636	85.45	61.82	71.74	78.93
plasma versican + plasma exosomal versican	0.4909	0.561	0.804	0.7343–0.8740	80.00	69.09	74.59	75.29

### Diagnostic performance of lung cancer markers NSE, CEA, CYFRA21-1, SCC, plasma versican, and plasma exosomal versican in NSCLC patients with metastasis

**Fig. 7:**
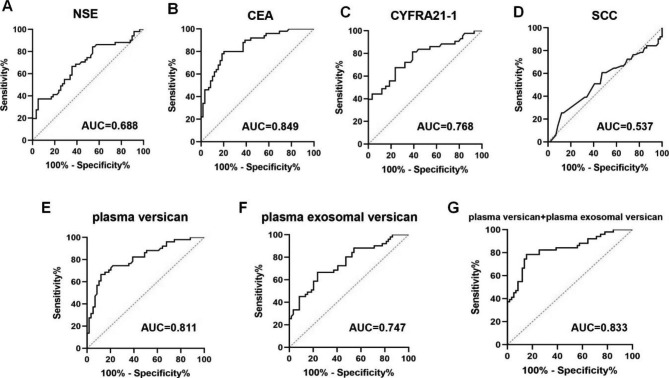
Diagnostic performance of lung cancer markers NSE, CEA, CYFRA21-1, SCC, plasma versican, and plasma exosomal versican in advanced-stage NSCLC patients

We further investigated whether the diagnostic performance of plasma versican and plasma exosomal versican was greater than other common lung cancer markers NSE, CEA, CYFRA21-1, and SCC in NSCLC patients with metastasis. As shown in Fig. 7A–G, when the cutoff value was 1885.00 pg/ml, the AUC of plasma versican was 0.811, and the sensitivity, specificity, PPV, and NPV was 68.63%, 84.75%, 79.55%, and 75.76%, respectively. Similarly, when the cutoff value was 234.70 pg/ml, the AUC of plasma exosomal versican was 0.747, and the sensitivity, specificity, PPV, and NPV was 66.67%, 76.27%, 70.83%, and 72.58%, respectively. When the threshold was 0.43, the AUC of the plasma versican + plasma exosomal versican was 0.833, and the combined detection sensitivity, specificity, PPV, and NPV was 78.43%, 84.75%, 81.64%, and 81.97%, respectively (Table 3).

**Table 3:** Diagnostic performance of lung cancer markers, plasma versican, and plasma exosomal versican in NSCLC patients with metastasis

Indicators	Youden index	Cutoff	AUC	AUC 95%CI	Sensitivity	Specificity	PPV(%)	NPV(%)
NSE	0.3217	15.49	0.688	0.5872–0.7880	37.25	94.92	86.37	63.64
CEA	0.5936	6.025	0.849	0.7782–0.9205	78	81.36	78.34	81.05
CYFRA21-1	0.4371	2.520	0.767	0.6721–0.8632	67.44	76.27	71.07	73.05
SCC	0.1332	0.810	0.537	0.4261–0.6470	60.78	52.54	52.54	60.78
plasma versican	0.5338	1885	0.811	0.7292–0.8919	68.63	84.75	79.55	75.76
plasma exosomal versican	0.4294	234.7	0.747	0.6548–0.8383	66.67	76.27	70.83	72.58
plasma versican + plasma exosomal versican	0.6318	0.428	0.833	0.7546–0.9110	78.43	84.75	81.64	81.97

It should be emphasized that the diagnostic value of CEA and CYFRA21-1 was similarly high among NSCLC patients with metastasis, which may have been due to the large number of stage IV patients who were recruited in this study. Following the results in Fig. 7; Table 3, the AUC values of plasma versican and plasma exosomal versican were found to be higher than those of NSE or SCC, and the diagnostic performance was found to be larger than that of NSE or SCC; nevertheless, they were comparable to those of CEA and CYFRA21-1. However, in NSCLC patients with metastasis, the AUC value of combined detection was higher, and the diagnostic performance was better.

### Risk assessment of plasma versican and plasma exosomal versican in predicting NSCLC or predicting metastasis in NSCLC patients

**Fig. 8:**
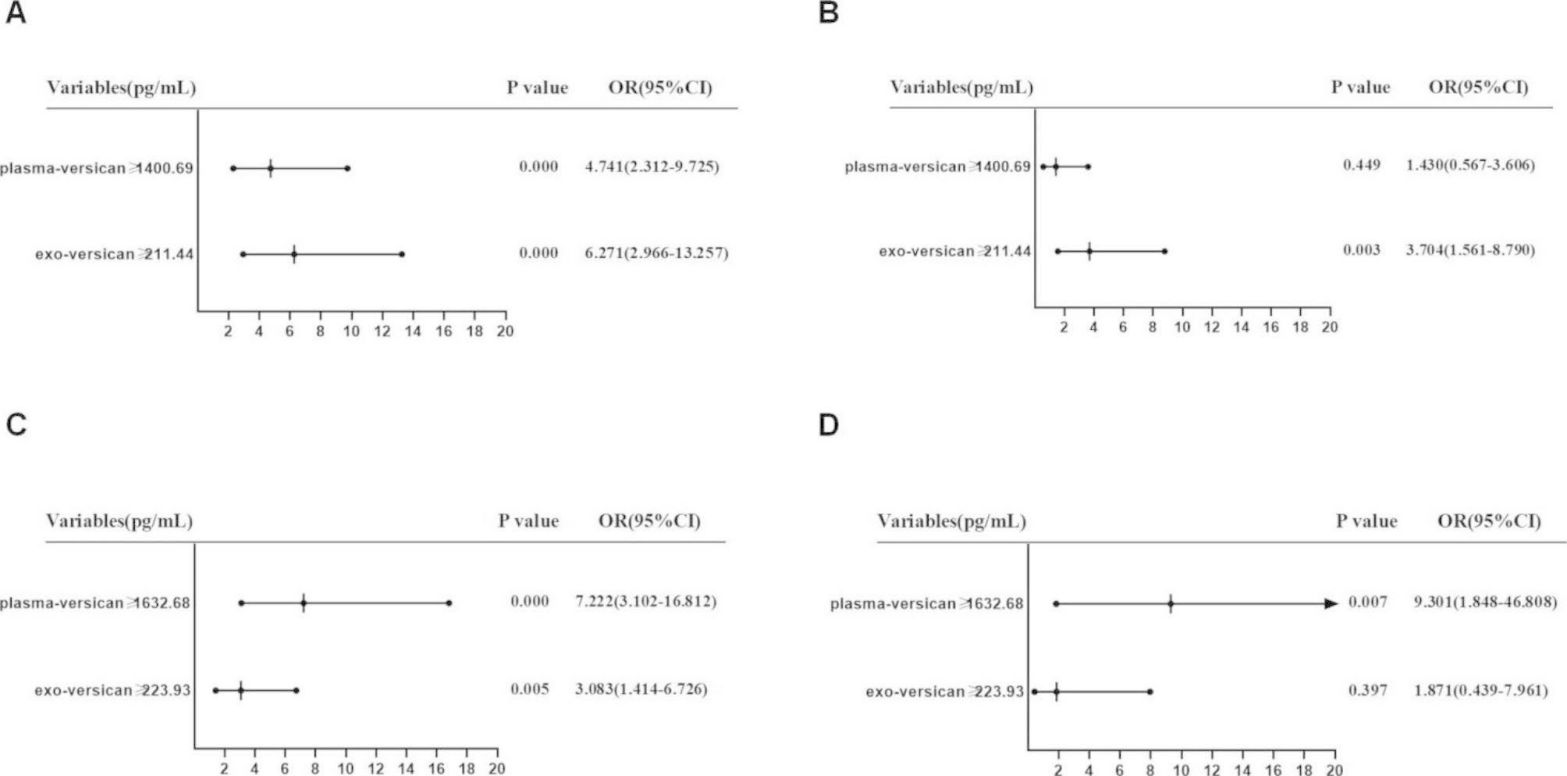
Risk assessment of plasma versican and plasma exosomal versican in predicting NSCLC or predicting metastasis in NSCLC patients.(**A,B**)The forest plot of Univariate and Multivariate logistic regression analyzes the Risk assessment in predicting NSCLC, multifactorial correction included the following variables: NSE, CEA, CYFRA21-1, SCC. (**C,D**)The forest plot of Univariate and Multivariate logistic regression analyzes the Risk assessment in predicting metastasis in NSCLC. multifactorial correction included the following variables: NSE, CEA, CYFRA21-1, SCC

When comparing the levels of plasma versican and plasma exosomal versican in all patient groups, we used binary logistic regression analysis to determine the predictive risk value of NSCLC. The median (two classifications) was evaluated as cutoff points. All patients were divided into a low-level group and a high-level group according to the median values of plasma versican (1400.69 pg/ml) and plasma exosomal versican (211.44 pg/ml). Compared with low plasma versican, the risk of NSCLC with high plasma versican was 4.741 (95%CI 2.312–9.725, *P* < 0.001), and the adjusted OR was 1.430 (95%CI 0.567–3.606, *P* > 0.05); Similarly, compared with low plasma exosomal versican, the risk of NSCLC with high plasma exosomal versican was 6.271 (95%CI 2.966–13.257, *P* < 0.001), and the adjusted OR was 3.704 (95%CI 1.561–8.790, *P* = 0.003) (Fig. 8A **and B** ).

In the NSCLC group, we used binary logistic regression analysis to analyze the predictive risk value of lung cancer metastasis by plasma versican and plasma exosomal versican levels. NSCLC patients were split into two groups according to the median values of plasma versican (1632.68 pg/ml) and plasma exosomal versican (223.93 pg/ml). Compared with low plasma versican, the risk of NSCLC with high plasma versican was 7.222 (95%CI 3.102–16.812, *P* < 0.001) and the adjusted OR was 9.301 (95%CI 1.848–46.808, *P* = 0.007). Similarly, compared with low plasma exosomal versican, the risk of NSCLC with high plasma exosomal versican was 3.083 (95%CI 1.414–6.726, *P* = 0.005), and the adjusted OR was 1.871 (95%CI 0.439–7.961, *P* > 0.05) (Fig. 8C **and D** ).

## Discussion

Many types of living cells release exosomes, which may be found in the intercellular space, extracellular fluid, or peripheral blood circulation. Exosomes are thought to have a significant role in disease development [22]. Exosome detection, a novel liquid biopsy technology, provides several benefits over existing methods, including ease of collection, non-invasiveness, and real-time dynamic detection [23]. Exosome proteins are persistent in the plasma of patients and exhibit distinct biological properties of tumor cells, making them a simple and reliable biomarker. Exosome proteins are also inexpensive and easy to obtain. As a result, exosomes may be useful in the early detection and assessment of prognosis in many different types of malignant tumors. A recent study performed a comprehensive proteomic analysis of extracellular vesicles and particles (EVPs) from human samples and discovered that versican was highly enriched in EVPs derived from explants of pancreatic and lung adenocarcinoma, suggesting that versican in extracellular vesicles may also serve as a tissue-specific EVP marker in addition to its other functions [24].

There are some lung cancer biomarker candidates that are currently being tested in large cohorts, such as seven autoantibodies (p53, NY-ESO-1, CAGE, GBU4-5, SOX2, HuD, and MAGE A4), complement fragments C4d, microRNAs, circulating tumor DNA, DNA methylation(SOX2 and PTGER4 methylation), blood protein analysis or RNA airway or nasal labeling [25]. In our study, we investigated for the first time the quantities of versican expressed in human plasma and plasma exosomes. Our findings indicated that versican was highly expressed in plasma and plasma exosomes from NSCLC patients, and that this expression was significantly associated with the clinicopathological features of lymph node metastasis and distal metastasis in NSCLC patients, as well as with the presence of a mutation. In addition, we evaluated the diagnostic value of plasma versican or plasma exosomal versican in patients with NSCLC, and the findings revealed that both were superior to that of NSE, CEA, CYFRA21-1, and SCC in the diagnosis of NSCLC, and that they were superior to that of NSE and SCC in the diagnosis of NSCLC with metastasis. These findings suggested that plasma versican or plasma exosomal versican had high clinical utility in the diagnosis and prediction of metastases in NSCLC.

Versican was shown to be overexpressed in a variety of proliferative cancers as well as tumor-associated stroma in several studies. The expression of versican V1 in hepatocellular carcinoma was high, and the EGF-like motif of versican V1 was found to promote the Warburg effect and cell proliferation by activating the EGFR–PI3K–AKT axis [26]. The expression of versican has been related to invasion and progression in malignant mixed tumors, such as carcinoma in mixed tumors (CMT) of the canine mammary gland. In addition, its interaction with surface cell receptors EGFR, human epidermal growth factor receptor 2(HER-2) and CD44 in malignant epithelial cells may be responsible for proliferation and cellular motility in early stages of cancer [27]. Versican V1 was also found to promote the Warburg effect and cell proliferation in other cancers, including breast cancer [28]. Versican is involved in the process of tumor cell metastasis and invasion in the tumor microenvironment, which is regulated by several different pathways. In ovarian cancer, for example, the expression of versican in cancer-associated fibroblasts is regulated and significantly upregulated by TGF-beta receptor II and the SMAD signaling pathway. Versican can promote tumor cell invasion and metastasis by activating NF-κB signaling and upregulating the hyaluronic acid receptor CD44, receptor for HA mediated motility(RHAMM), and matrix metalloproteinase 9 (MMP9) [29]. Using a mouse model of Lewis lung cancer, we were able to demonstrate that versican and its cleavage products from matrix play an important role in the tumor microenvironment [30]. In addition to promoting angiogenesis and tumor growth, the microenvironment can also affect versican expression in tumor cells. Versican has been shown to have a role in the promotion of angiogenesis in malignant tumors by interacting with fibronectin, vascular endothelial growth factor(VEGF), HA, and other molecules in the tumor microenvironment. However, the mechanism by which it does this and the signaling pathway that it uses are still being investigated.

Until recently, IHC was the most frequently used technique for detecting versican expression in tumor tissues. Versican has significant clinical importance in the diagnosis, prognosis, and recurrence of diverse malignant tumors. Using gene expression profiling and IHC of tissue microarrays, it was demonstrated that versican was associated with the clinicopathological characteristics of gastric cancer and that it could be used as an independent adverse prognostic index for patients with non-metastatic gastric cancer [7]. Versican expression is related to poor prognosis in hepatocellular carcinoma, including increased tumor-associated macrophages(TAMs) infiltration, poor tumor differentiation, and TNM stage [31]. Versican and its related components were also shown to be possible diagnostic indicators for multiple myeloma [32]. In our study, versican in plasma and plasma exosomes was evaluated using ELISA and shown to be linked to TNM stage, lymph node metastasis, distant metastasis, and mutation (all *P* < 0.05). The early diagnosis ROC curve of plasma versican and plasma exosomal versican had better performance than the ROC curves for NSE, CEA, CYRFA21-1, and SCC, and they also performed better in the diagnosis of metastatic lung cancer. Combined detection performed better in the diagnosis of NSCLC with metastasis than individual detection alone. The fact that CEA was more successful in the detection of lung cancer metastasis, which may be connected to the high proportion of T3 + T4 stage patients in our research, should be noted.

Our results suggested that plasma exosomal versican may also be useful in early NSCLC risk assessment. The risk of NSCLC in the high plasma exosomal versican group was still higher than that in the low-level group when the median was the cutoff point (*P* < 0.05), but there was no difference between the two groups of plasma versican. The results suggested that when plasma exosomal versican ≥ 211.44 pg/ml, the risk of NSCLC was higher, indicating that the value and significance of detecting plasma exosomal protein was greater in the early diagnosis of lung cancer and might be related to the envelopment of exosomes that made the versican protein more stable. Interestingly, plasma versican seemed to have better predictive value in the risk assessment of NSCLC metastasis. When the median was used the cutoff point, the risk of NSCLC metastasis in the high plasma versican group was higher than that in the low level group (*P* < 0.05), but there was no difference between the two groups of plasma exosomal versican. The results showed that the risk of NSCLC metastasis was higher when plasma versican ≥ 1632.68 pg/ml, suggesting that in the advanced stage of lung cancer, versican secreted by lung cancer will enter the peripheral blood in large quantities, and the detection of plasma versican protein will have more clinical value.

In conclusion, our work first evaluated versican expression in plasma and plasma exosomes of NSCLC patients, and then investigated the clinical diagnostic significance of versican expression in these patients. There are, however, certain limitations that must be considered. First, we used “matched” controls for non-NSCLC cases in this study, which may have given us a better diagnostic value. Furthermore, the overall sample size was small, and we didn’t classify smokers based on pack-year history, which maybe have had an impact on lung cancer risk and possibly versican levels. Additionally, the small sample size of the control group did not accurately represent the universal significance of plasma versican in large clinical samples. As a result, the value of versican as a novel tumor marker for NSCLC has not yet been thoroughly assessed. To further confirm the clinical significance of versican in NSCLC patients and other tumors, validation of the detection of versican in large prospective cohorts is still needed in the future. However, more basic research is required to explore the potential molecular mechanism of versican, particularly exosomal versican.

## Electronic supplementary material

Below is the link to the electronic supplementary material.

Supplementary Material 1

## Data Availability

The data used to support the findings of this study are included within the article. Further enquiries can be directed to the corresponding authors.

## Abbreviations

NSCLC: Non-small cell lung cancer
NSE: Neuron-specific enolization enzyme
CEA: Carcinoembryonic antigen
CYFRA21-1: Cytokeratin 19
SCC: Squamous cell carcinoma antigen.
