# Supplementary material for: Plasma versican and plasma exosomal versican as potential diagnostic markers for non-small cell lung cancer
Source: Respir Res. 2023 May 31;24:140. doi: 10.1186/s12931-023-02423-4 (PMC10230736; doi:10.1186/s12931-023-02423-4)
Supplement: Supplementary file 1 — Supplementary Material 1 [file 12931_2023_2423_MOESM1_ESM.pdf]

original, full-length gel and blot images for exosome identification

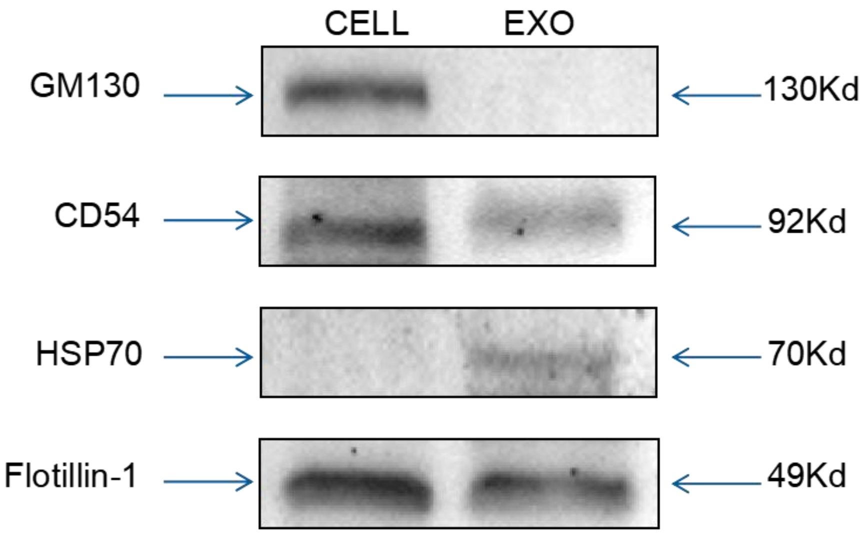

A

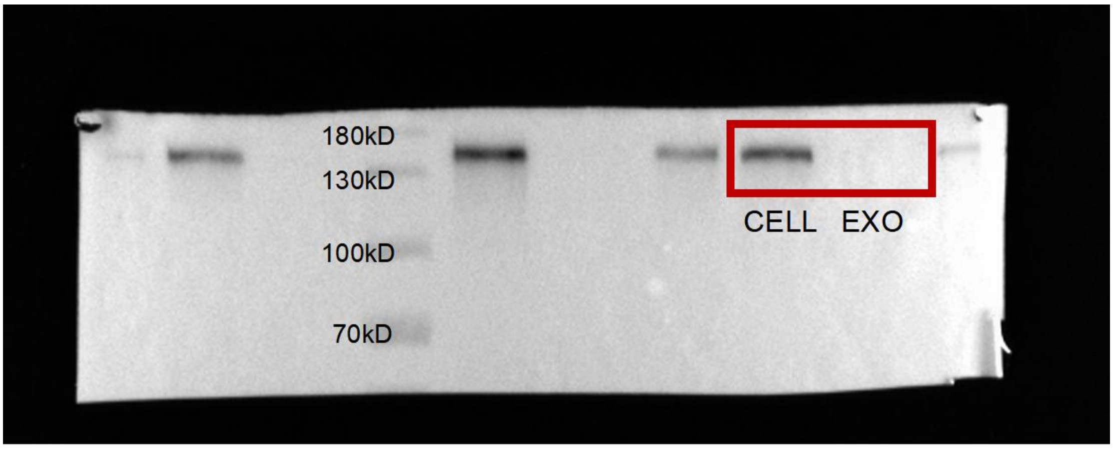

B

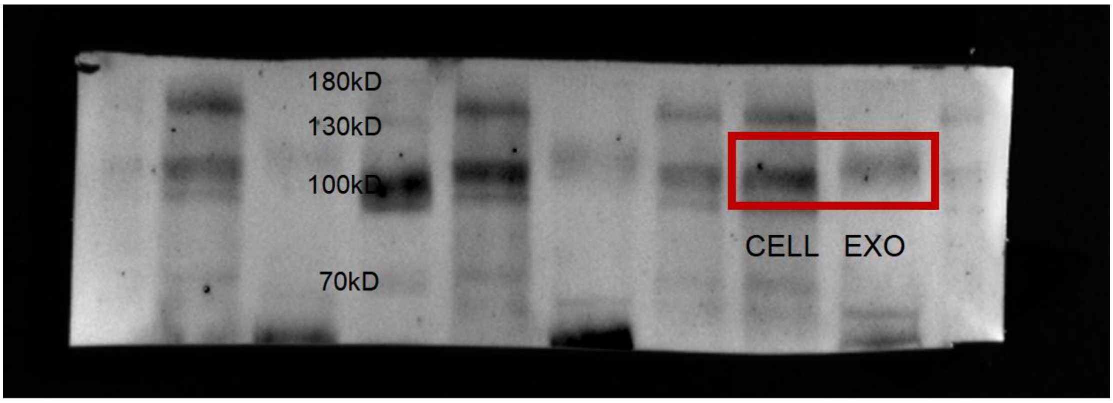

C

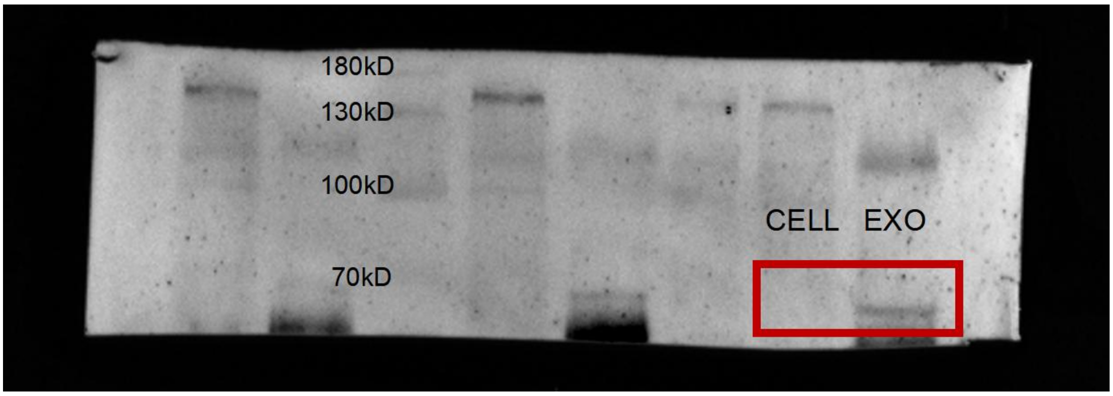

D

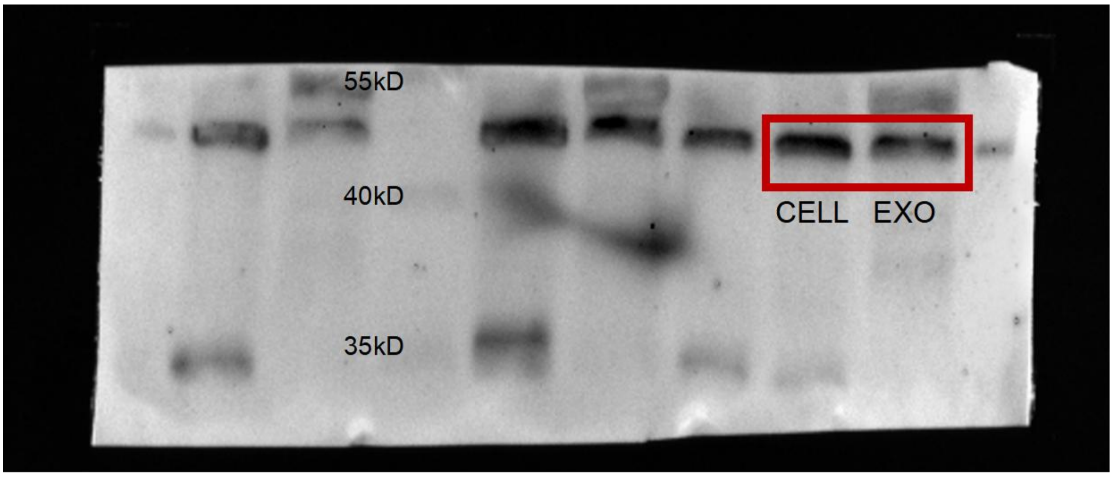

Supplement Figure.1 Western blotting analysis of characteristic markers of exosomes, including CD54(A), HSP70(B), and Flotillin-1(C), with GM130(D) as a negative control protein.
